# Association of Maternal Age With Severe Maternal Morbidity and Mortality in Canada

**DOI:** 10.1001/jamanetworkopen.2019.9875

**Published:** 2019-08-23

**Authors:** Kazuyoshi Aoyama, Ruxandra Pinto, Joel G. Ray, Andrea D. Hill, Damon C. Scales, Stephen E. Lapinsky, Michelle A. Hladunewich, Gareth R. Seaward, Robert A. Fowler

**Affiliations:** 1 Department of Anesthesia and Pain Medicine, The Hospital for Sick Children, Toronto, Ontario, Canada; 2 Program in Child Health Evaluative Sciences, SickKids Research Institute, Toronto, Ontario, Canada; 3 Institute of Health Policy, Management and Evaluation, University of Toronto, Toronto, Ontario, Canada; 4 Department of Critical Care Medicine, Sunnybrook Health Science Center, Toronto, Ontario, Canada; 5 The Keenan Research Centre of The Li Ka Shing Knowledge Institute of St Michael’s Hospital, Toronto, Ontario, Canada; 6 Department of Obstetrics and Gynecology, St Michael’s Hospital, Toronto, Ontario, Canada; 7 Department of Critical Care Medicine, Mount Sinai Hospital and University Health Network, Toronto, Ontario, Canada; 8 Kidney Care Centre, Sunnybrook Health Science Center, Toronto, Ontario, Canada; 9 Division of Maternal-Fetal Medicine, Department of Obstetrics & Gynaecology, Mount Sinai Hospital, Toronto, Ontario, Canada

## Abstract

**Importance:**

Over the past 2 decades, there has been a trend toward increasing maternal age in many high-income countries. Maternal age may lead to greater attendant morbidity and mortality for Canadian mothers.

**Objective:**

To investigate the association of maternal age, adjusting for patient-level and hospital-level factors, with severe maternal morbidity (SMM) and maternal death in Canada.

**Design, Setting, and Participants:**

A nationwide population-based cohort study of all antepartum, peripartum, and postpartum women and adolescents seen at Canadian acute care hospitals from April 1, 2004, to March 31, 2015. All analyses were completed on September 13, 2018.

**Exposures:**

Maternal age at the index delivery.

**Main Outcomes and Measures:**

Severe maternal morbidity and maternal death during pregnancy and within 6 weeks after termination of pregnancy.

**Results:**

During the study period, there were 3 162 303 new pregnancies (mean [SD] maternal age, 29.5 [5.6] years) and 3 533 259 related hospital admissions. There were 54 219 episodes of SMM (17.7 cases per 1000 deliveries) in the entire study period, with a 9.8% relative increase from 2004-2005 to 2014-2015, in addition to an increasing proportion of pregnancies to older mothers. Independent patient-level factors associated with SMM included increasing Maternal Comorbidity Index; maternal age 19 years or younger and 30 years or older, with the greatest risk experienced by women 45 years or older (odds ratio [OR], 2.69; 95% CI, 2.34-3.06 compared with maternal age 20-24 years); and lowest income quintile (OR, 1.19; 95% CI, 1.14-1.22 compared with highest income quintile). Hospital-level factors associated with SMM included specific provinces. Independent patient-level factors associated with maternal mortality included increasing Maternal Comorbidity Index, age 40 to 44 years (OR, 3.39; 95% CI, 1.68-6.82 compared with age 20-24 years), age 45 years or older (OR, 4.39; 95% CI, 1.01-19.10 compared with age 20-24 years), and lowest income quintile (OR, 4.14; 95% CI, 2.03-8.50 compared with highest income quintile). Hospital-level factors associated with maternal mortality included lowest hospital pregnancy volume.

**Conclusions and Relevance:**

In Canada, maternal age and SMM have increased over the past decade. Results of this study suggest that province of residence, maternal comorbidity, residence income quintile, and extremes of maternal age, especially those 45 years or older, were associated with SMM and mortality. These findings are relevant to prospective parents, their health care team, and public health planning.

## Key Points

**Question:**

Is maternal age associated with severe maternal morbidity and with maternal death in Canada?

**Findings:**

In this nationwide population-based cohort study of 3.1 million pregnancies in Canada, severe maternal morbidity has increased over the past decade, and this trend coincided with an increase over time in maternal age and in the proportion of pregnancies to older mothers. Extremes of maternal age, especially those 45 years or older compared with those aged 20 to 24 years, were associated with severe maternal morbidity and with maternal mortality.

**Meaning:**

Increasing maternal age was an independent characteristic associated with severe maternal morbidity and mortality.

## Teaser

This nationwide population-based cohort study investigates the association of maternal age, adjusting for patient-level and hospital-level factors, with severe maternal morbidity and maternal death in Canada.

## Introduction

The World Health Organization estimates that more than 300 000 women die each year from pregnancy or childbirth-related complications.^1^ The Global Strategy for Women’s, Children’s and Adolescents’ Health (2016-2030), seeks to end all preventable deaths of women, children, and adolescents.^1^ While maternal mortality has decreased in the developed world over the past 25 years,^2^ a recent nationwide cohort study found that severe maternal morbidity (SMM) in Canada may be increasing.^3,4^ The latest rate of SMM in Canada is around 17 cases per 1000 deliveries.^3^

In many high-income countries, adolescent (age range, 10-19 years) birth rates are declining, whereas there has been a trend toward increasing maternal age.^5^ Hence, more attention has shifted toward ascertaining adverse outcomes in older mothers (≥35 years). Older maternal age is associated with increased childbirth-related complications.^6,7,8,9^ However, little information is available on associations of increasing maternal age with SMM and maternal mortality, especially in the Canadian context.

Therefore, we performed a nationwide population-based cohort study of pregnancies in Canada. We explored trends in maternal age and the association between maternal age and outcomes for pregnant and postpartum women and adolescents.

## Methods

### Study Design, Data Sources, and Population

We conducted an 11-year nationwide population-based cohort study using data derived from the Discharge Abstract Database (DAD) of the Canadian Institute for Health Information (CIHI). The DAD captures administrative, clinical, and demographic information on all acute hospital discharges across Canada, except for the province of Quebec.^10,11^ This study was conducted under the data security and privacy policy of the CIHI and was approved by the institutional review boards at the University of Toronto and Mount Sinai Hospital; patient informed consent was not required because all data were deidentified. This study followed the Strengthening the Reporting of Observational Studies in Epidemiology (STROBE) reporting guideline.

All pregnant and postpartum (within 6 weeks of delivery) women and adolescents, including those with ectopic pregnancy or spontaneous abortion, seen at acute care hospitals from April 1, 2004, to March 31, 2015, were included in this nationwide population-based cohort study. All analyses were completed on September 13, 2018. Variables included maternal demographics (age; comorbid conditions as classified by the *International Statistical Classification of Diseases and Related Health Problems, Tenth Revision, Canada* [*ICD-10CA*]; socioeconomic status; and residence location), pregnancy-specific variables (gestational week at admission and parity), hospital-level and system-level variables (province, territory, and urban vs rural location), and outcome-related variables (vital status at hospital discharge, transfers between hospitals, and readmissions) (eTable 1 in the Supplement). A previously described algorithm captures all hospital admissions and discharges over a single pregnancy as an episode of care.^3^ When more than 1 admission per episode existed, variables of the first admission of the episode were used.

### Exposures

The main exposure of interest was maternal age at the index delivery. Maternal age was categorized into 5-year age bins and used as a categorical variable in the analyses.

### Outcomes

The primary outcome of this study was SMM during an episode of care (pre–life-threatening condition) defined by the Canadian Perinatal Surveillance System.^12^ Severe maternal morbidity originally consisted of 34 conditions (eg, eclampsia and postpartum hemorrhage requiring transfusion), diagnosed with the *ICD-10CA* and the *Canadian Classification of Health Interventions*. In this study, HIV and blood transfusion were removed from the list because these do not reflect life-threatening conditions.^3^ Also, chronic congestive heart failure and preexisting hypertension and renal disease were removed from the list because these conditions were not acute SMM.

Maternal death (during pregnancy and within 6 weeks after termination of pregnancy) was the secondary outcome. This was determined based on either the *ICD-10CA* codes or death as vital status at hospital discharge.

### Statistical Analysis

Descriptive statistics included counts and proportions, means and standard deviations, and medians and interquartile ranges, as appropriate. First, we calculated relative changes over the entire study period by comparing the rates in 2014-2015 relative to 2004-2005 based on the following equation: [(rates in 2014-2015 − rates in 2004-2005) / (rates in 2004-2005)] × 100. Second, we used standardized differences to compare the characteristics of patients who did or did not develop SMM.^13^ Third, the number needed to be exposed for 1 additional person to be harmed was calculated for each SMM indicator (ie, the number of women and adolescents with this condition needed to result in 1 additional death).^14^ Fourth, we examined the association between maternal age and SMM and the association between maternal age and maternal death independently using a multilevel logistic regression model with random intercepts to account for clustering of patients within hospitals, which generated odds ratios (ORs) and 95% CIs.^15^

Patient-level covariates included age, parity, socioeconomic status by household postal code income quintile, urban or rural residence location, and whether the patient was transferred or not between hospitals. Hospital-level covariates included province or territory, urban or rural hospital location, and hospital pregnancy volume.^16^ We used the Maternal Comorbidity Index to perform additional risk adjustment among mothers with different baseline characteristics in all models.^17^ The original Maternal Comorbidity Index consisted of 19 comorbidities (eg, drug abuse, sickle cell disease, and chronic congestive heart failure). Maternal age was removed from the Maternal Comorbidity Index because age range of the Maternal Comorbidity Index does not include younger than 35 years, but age and the Maternal Comorbidity Index were separately entered into the model. The Maternal Comorbidity Index was eventually categorized into 3 groups (0, 1, and ≥2). Because 6 conditions of the Maternal Comorbidity Index had potential overlap with SMM outcome conditions, only preexisting conditions were included in the Maternal Comorbidity Index, and those occurring after pregnancy onset were considered as outcomes for SMM to ensure a valid temporal association.^3,17^ We used listwise deletion for records with missing data and compared characteristics of women and adolescents with missing and nonmissing data to ensure generalizability.^18^

### Sensitivity Analyses

We repeated the models for primary and secondary outcomes by exploring hospital pregnancy volume as a function of the number of pregnancy-related intensive care unit (ICU) admissions and, separately, the pregnancy-related ICU admission rate at each hospital (the numerator is the number of ICU admissions, and the denominator is the number of pregnancy admissions, both over the entire study period).

Three post hoc sensitivity analyses were performed. First, we restricted the cohort of analysis to the first pregnancy episode experienced by a woman or adolescent because our primary analysis was done in the cohort that included more than 1 pregnancy episodes experienced by the same woman or adolescent. Second, because of a high missing rate in income quintile, we attempted to confirm findings using listwise deletion for records with missing data by a different approach. To address data missing at random for 3 variables—income quintile (13.9% missing), residence rurality (0.8%), and hospital rurality (0.6%)—all analyses were redone with 20 multiple imputation data sets using fully conditional specification and discriminant function methods to impute these categorical values, which affords good efficiency of the coefficients (ie, smaller standard error of the point estimates) and sufficient power to detect differences.^19,20,21,22^ Third, because we wanted to explore the association of hospital-to-hospital transfer status on SMM and because SMM could occur before a patient was transferred from one hospital to another, we excluded from the cohort those who had SMM and for whom there was a transfer from one hospital to another that occurred on the first hospital admission during an episode of pregnancy.

All analyses were performed using SAS statistical software (version 9.4; SAS Institute Inc) and Excel for Macintosh (version 15.3.9; Microsoft Corp). All statistical tests were 2-sided, and *P* < .05 was considered statistically significant.

## Results

### Incidence, Clinical Characteristics, and Outcomes of Pregnancies in Canada

During the study period, there were 3 162 303 new pregnancies (mean [SD] maternal age, 29.5 [5.6] years) and 3 533 259 related hospital admissions among 2 035 453 mothers, resulting in 3 026 323 live births, 29 067 stillbirths, and 106 913 abortions or ectopic pregnancies. There were 54 219 episodes of SMM (17.7 cases per 1000 deliveries among 3 055 390 live births and stillbirths) in the entire study period (Table 1 and Table 2). In this study, 2.4% of all SMM events happened during the antepartum period, including ectopic pregnancies or abortions. Women and adolescents who developed SMM had a higher preexisting Maternal Comorbidity Index (Table 1). The most common reasons underlying SMM included postpartum hemorrhage requiring blood products (5.5 cases per 1000 deliveries), sepsis (3.8 cases per 1000 deliveries), and cardiac failure (1.5 cases per 1000 deliveries) (eTable 2 in the Supplement). The most common causes of SMM and the accompanying number needed to have that diagnosis for 1 additional person to die are listed in eTable 3 in the Supplement. Respiratory and cardiac illnesses among pregnant and postpartum women and adolescents were associated with the greatest individual risk of harm; however, other conditions, such as sepsis and hemorrhage, were highly prevalent. Postpartum hemorrhage requiring transfusion and sepsis were the first and second most common SMM across all ages, but the rate varied depending on age group (eTable 4 in the Supplement). In adolescents (age range, 10-19 years), the third most common SMM was eclampsia. In older mothers (≥35 years), the third most common SMM was cardiac failure, with the highest rate (2.3 cases per 1000 deliveries) compared with other age groups.

**Table 1.** Baseline Characteristics of Patients With and Without SMM

Variable	No. (%)		
	SMM (n = 55 228)	No SMM (n = 3 107 075)	Standardized Difference^a^
Delivery admission	52 471 (95.9)	2 949 718 (95.8)	0.00
Antepartum admission	10 408 (19.0)	240 276 (7.8)	0.33
Postpartum admission	15 697 (28.7)	44 995 (1.5)	0.82
Abortion or ectopic pregnancy–related admission	1009 (1.8)	105 904 (3.4)	−0.10
Age group, mean (SD), y	29.9 (6.1)	29.5 (5.6)	0.08
10-14	44 (0.1)	1059 (0.0)	0.15
15-19	2842 (5.1)	129 506 (4.3)	
20-24	7871 (14.3)	458 984 (15.3)	
25-29	14 044 (25.9)	876 504 (29.1)	
30-34	16 568 (30.6)	964 336 (32.0)	
35-39	9964 (18.5)	471 591 (15.9)	
40-44	2589 (4.9)	93 058 (3.2)	
≥45	289 (0.6)	5039 (0.2)	
Gestational week at delivery admission, mean (SD), wk	37.6 (3.8)	38.7 (2.5)	−0.34
Maternal Comorbidity Index			
0	38 814 (68.2)	2 533 388 (82.5)	0.41
1	8651 (17.3)	415 697 (13.4)	
≥2	7255 (14.5)	130 179 (4.1)	
Live birth	47 747 (95.7)	2 978 576 (95.9)	0.00
Singleton	45 575 (91.3)	2 930 345 (94.3)	−0.11
Twin	2164 (4.3)	49 306 (1.6)	0.16
≥Triplet	95 (0.2)	1691 (0.0)	0.04
Parity			
0	31 239 (62.6)	1 801 520 (58.0)	0.10
1	10 800 (21.6)	808 488 (26.0)	
≥2	7850 (15.7)	497 351 (16.0)	
Residence, 0.8% missing (n = 24 496)			
Urban	44 114 (80.6)	2 523 874 (82.0)	−0.03
Rural	10 606 (19.4)	2 523 874 (18.0)	
Income quintile, 13.9% missing (n = 439 560)			
1, Lowest quintile	113 238 (24.2)	671 113 (24.2)	0.06
2	10 078 (18.4)	560 051 (18.2)	
3	8847 (16.2)	511 702 (16.6)	
4	8059 (14.7)	482 679 (15.7)	
5, Highest quintile	7226 (13.2)	425 877 (13.8)	
Hospital, 0.6% missing (n = 19 420)			
Urban	41 407 (75.7)	2 368 309 (76.9)	−0.03
Rural	13 282 (24.3)	709 951 (23.1)	
Delivery mode in delivery admission			
Vaginal	16 786 (30.7)	2 123 604 (69.0)	−0.82
Cesarean delivery	20 907 (38.2)	793 245 (25.8)	0.27
Discharge for all admission episodes			
Home	51 761 (94.6)	3 019 533 (98.1)	0.19
Transfer	421 (0.8)	3536 (0.1)	
Province			
Newfoundland and Labrador	955 (1.9)	50 513 (1.6)	0.10
Prince Edward Island	252 (0.5)	15 169 (0.5)	
Nova Scotia	1456 (2.9)	94 324 (3.0)	
New Brunswick	1217 (2.4)	79 179 (2.5)	
Ontario	22 347 (44.8)	1 506 890 (48.5)	
Manitoba	3096 (6.2)	177 378 (5.7)	
Saskatchewan	2849 (5.7)	155 270 (5.0)	
Alberta	10 081 (20.2)	544 173 (17.5)	
British Columbia	7240 (14.5)	466 777 (15.0)	
Territories	396 (0.8)	17 686 (0.6)	

Abbreviation: SMM, severe maternal morbidity.

^a^ Standardized difference is the difference in means or proportions divided by the standard error. Standardized mean differences of 0.2, 0.5, and 0.8 are often or generally equated to effect sizes of small, medium, and large, respectively.

**Table 2.** Outcomes for Patients With and Without SMM

Variable	No. (%)		Standardized Difference^a^
	SMM (n = 55 228)	No SMM (n = 3 107 075)	
ICU admission	6099 (11.1)	3997 (0.1)	0.49
ICU	5694 (10.4)	3451 (0.1)	0.47
Step-up/step-down care unit	634 (0.1)	593 (0.0)	0.15
Length of ICU stay, mean (SD), h	85.5 (184.4)	90.5 (181.3)	−0.02
Length of hospital stay, mean (SD), d	6.9 (9.9)	2.6 (3.1)	0.58
Hospital mortality	178 (0.3)	14 (0.0)	0.08
ICU mortality in ICU admission	111 (0.2)	1 (0.0)	0.06
Groups according to hospital volume of pregnancy			
1, Lowest volume	1390 (2.8)	83 174 (2.7)	0.13
2	4548 (9.1)	301 798 (9.7)	
3	16 227 (32.5)	106 2977 (34.2)	
4	13 638 (27.3)	957 520 (30.8)	
5, Highest volume	14 086 (28.2)	701 890 (22.6)	

Abbreviations: ICU, intensive care unit; SMM, severe maternal morbidity.

^a^ Standardized difference is the difference in means or proportions divided by the standard error. Standardized mean differences of 0.2, 0.5, and 0.8 are often or generally equated to effect sizes of small, medium, and large, respectively.

### SMM and Increasing Maternal Age in Canada

The lowest incidence of SMM was among women in their twenties, with greater morbidity among younger and older mothers (Figure and Table 3). Severe maternal morbidity increased over the study period (a 9.8% relative increase) from 17.2 cases per 1000 deliveries in 2004-2005 to 18.9 cases per 1000 deliveries in 2014-2015, corresponding to an increasing proportion of pregnancies in older (≥40 years) women in Canada over the study period (Figure).

**Figure.**
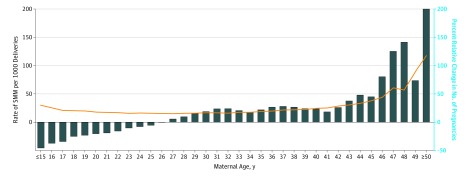
Rate of Severe Maternal Morbidity (SMM) Over the Entire Study Period and Percent Relative Change in the Number of Pregnancies, by Maternal Age, Between 2004-2005 and 2014-2015 The line shows the rate of SMM. The bars show the percent relative change in the number of pregnancies.

**Table 3.** Temporal Trends in Severe Maternal Morbidity by Maternal Age

Age Group, y	No. (Rate per 1000 Deliveries)											
	2004-2005	2005-2006	2006-2007	2007-2008	2008-2009	2009-2010	2010-2011	2011-2012	2012-2013	2013-2014	2014-2015	Total
10-14	NR	7 (55.1)	NR	NR	NR	9 (75.6)	NR	NR	NR	NR	NR	45 (37.1)
15-19	250 (19.0)	275 (21.0)	272 (19.6)	297 (20.8)	309 (21.7)	297 (21.5)	254 (19.7)	260 (21.9)	253 (22.6)	215 (21.7)	202 (21.3)	2884 (20.9)
20-24	771 (17.2)	766 (16.9)	664 (14.1)	768 (16.0)	772 (16.3)	689 (14.9)	731 (16.8)	705 (16.8)	746 (18.4)	709 (18.0)	688 (17.8)	8009 (16.6)
25-29	1247 (15.8)	1246 (15.6)	1125 (13.8)	1257 (14.8)	1264 (14.8)	1309 (15.1)	1287 (15.2)	1341 (15.8)	1452 (17.2)	1345 (16.3)	1403 (16.8)	14 276 (15.6)
30-34	1378 (16.3)	1401 (16.4)	1337 (15.4)	1345 (15.0)	1470 (16.2)	1474 (16.2)	1506 (16.5)	1595 (16.9)	1710 (17.7)	1799 (18.2)	1828 (17.9)	16 843 (16.7)
35-39	761 (19.0)	825 (20.0)	806 (18.5)	849 (18.6)	934 (20.4)	920 (19.8)	975 (20.9)	968 (20.7)	1017 (21.2)	1020 (21.1)	1111 (22.1)	10 186 (20.3)
40-44	208 (25.3)	236 (27.7)	166 (19.5)	213 (24.2)	215 (23.6)	231 (24.6)	256 (26.0)	279 (27.6)	309 (30.4)	272 (26.4)	294 (28.6)	2679 (26.0)
≥45	23 (57.9)	17 (43.7)	19 (41.0)	24 (49.0)	28 (54.3)	24 (41.0)	22 (38.7)	42 (65.1)	30 (48.0)	42 (63.0)	35 (50.4)	306 (50.7)
All ages	4641 (17.2)	4773 (17.4)	4394 (15.6)	4756 (16.3)	4997 (17.0)	4953 (16.8)	5036 (17.4)	5194 (17.9)	5519 (18.9)	5403 (18.6)	5562 (18.9)	55 228 (17.5)

Abbreviation: NR, not reported (cells with 1-5 counts of severe maternal morbidity were suppressed as per privacy policy of the Canadian Institute for Health Information).

Independent patient-level factors associated with SMM included increasing Maternal Comorbidity Index; maternal age 19 years or younger and 30 years or older, with the greatest risk experienced by women 45 years or older (OR, 2.69; 95% CI, 2.34-3.06 compared with maternal age 20-24 years); transfer between hospitals (OR, 2.01; 95% CI, 1.90-2.14); and lowest income quintile (OR, 1.19; 95% CI, 1.14-1.22 compared with highest income quintile) (Table 4). Hospital-level factors associated with SMM included specific provinces (OR, 0.78; 95% CI, 0.68-0.89 for British Columbia compared with Ontario) (Table 4 and eTable 5 in the Supplement).

**Table 4.** Odds Ratios for SMM and Mortality Occurring Within Pregnancy Course Using Multilevel Logistic Regression Models

Variable	Odds Ratio (95% CI)	
	SMM	Mortality
**Patient-Level Variables**		
Maternal Comorbidity Index		
0	1 [Reference]	1 [Reference]
1	1.62 (1.57-1.67)	1.70 (1.06-2.72)
≥2	3.67 (3.56-3.78)	10.20 (10.20-10.20)
Age group, y		
10-14	1.19 (0.78-1.79)	0.00 (0.00-0.00)
15-19	1.14 (1.08-1.20)	0.58 (0.20-1.68)
20-24	1 [Reference]	1 [Reference]
25-29	1.01 (0.98-1.04)	1.26 (0.75-2.14)
30-34	1.12 (1.08-1.15)	1.26 (0.74-2.14)
35-39	1.31 (1.27-1.36)	1.93 (1.11-3.39)
40-44	1.60 (1.52-1.68)	3.39 (1.68-6.82)
≥45	2.69 (2.34-3.06)	4.39 (1.01-19.10)
Parity		
0	1.30 (1.26-1.34)	1.60 (1.00-2.56)
1	0.91 (0.89-0.94)	1.23 (0.75-2.05)
≥2	1 [Reference]	1 [Reference]
Residence, urban vs rural	1.02 (0.99-1.05)	1.01 (0.65-1.57)
Transfer	2.01 (1.90-2.14)	11.00 (7.10-17.10)
Income quintile		
1, Lowest quintile	1.19 (1.14-1.22)	4.14 (2.03-8.50)
2	1.11 (1.07-1.15)	3.39 (1.63-7.03)
3	1.05 (1.01-1.08)	3.94 (1.90-8.08)
4	1.02 (0.98-1.05)	3.00 (1.42-6.36)
5, Highest quintile	1 [Reference]	1 [Reference]
**Hospital-Level Variables**		
Groups according to hospital volume of pregnancy		
1, Lowest volume	1.21 (1.01-1.46)	5.26 (1.73-15.96)
2	1.06 (0.92-1.23)	1.57 (0.52-4.71)
3	1.14 (1.01-1.30)	1 [Reference]
4	1.04 (0.91-1.17)	1.00 (0.47-2.12)
5, Highest volume	1 [Reference]	1.19 (0.59-2.36)
Province		
Newfoundland and Labrador	1.12 (0.89-1.40)	1.35 (0.50-3.63)
Prince Edward Island	1.07 (0.65-1.79)	NA
Nova Scotia	0.99 (0.78-1.27)	0.76 (0.28-2.08)
New Brunswick	0.92 (0.73-1.16)	0.37 (0.09-1.58)
Ontario	1 [Reference]	1 [Reference]
Manitoba	1.05 (0.86-1.28)	0.65 (0.29-1.46)
Saskatchewan	1.21 (1.01-1.46)	1.08 (0.54-2.18)
Alberta	1.16 (1.02-1.32)	0.75 (0.46-1.21)
British Columbia	0.78 (0.68-0.89)	0.48 (0.28-0.84)
Territories	1.72 (1.00-2.94)	NA
Hospital, urban vs rural	1.11 (0.95-1.28)	2.83 (0.65-12.30)

Abbreviations: NA, not applicable (because of no death or only 1 death during the study period, these were not included in the analyses); SMM, severe maternal morbidity.

### Maternal Mortality in Canada

During the study period, 192 mothers died, with a maternal mortality rate of 0.01%, or 6.2 per 100 000 deliveries (Table 2). Independent patient-level factors associated with maternal mortality included increasing Maternal Comorbidity Index, age 40 to 44 years (OR, 3.39; 95% CI, 1.68-6.82 compared with age 20-24 years), age 45 years or older (OR, 4.39; 95% CI, 1.01-19.10 compared with age 20-24 years), transfer between hospitals (OR, 11.00; 95% CI, 7.10-17.10), and lowest income quintile (OR, 4.14; 95% CI, 2.03-8.50 compared with highest income quintile) (Table 4). Hospital-level factors associated with maternal mortality included lowest hospital pregnancy volume and specific provinces (OR, 0.48; 95% CI, 0.28-0.84 for British Columbia compared with Ontario) (Table 4 and eTable 6 in the Supplement).

### Sensitivity Analyses

We performed sensitivity analyses by replacing quintile of hospital pregnancy volume with pregnancy-related ICU admission volume in one model and the rate in another model and found similar outcomes (eTable 7 in the Supplement). Restricting the cohort of analyses to the first pregnancy (57.9%), the incidence of SMM was 17.0 cases per 1000 deliveries; other findings did not change (eTable 8 in the Supplement). Using multiple imputation to explore the consequences of any missing data yielded similar findings (eTable 9 in the Supplement). Last, excluding those who had SMM before transfer at the first admission hospital during an episode (152 of 421 [36.1%] patients), findings were similar (eTable 10 in the Supplement).

## Discussion

In this nationwide population-based cohort study, we found that the incidence of SMM during the pregnancy and postpartum period increased between 2004-2005 and 2014-2015, and this trend coincided with an increase over time in maternal age and in the proportion of pregnancies to older mothers. Increasing maternal age was an independent characteristic of SMM and mortality throughout the study period.

### Other Studies

Recent population-based studies^3,4^ have demonstrated annual increases in SMM over the past decade in Canada of approximately 1.3% (95% CI, 0.6%-2.0%). The United States has experienced a similar trend in maternal age and SMM^23,24^; however, direct country-to-country comparisons are challenging due to differences in available data and definitions.^25,26^ Other jurisdictions have also reported higher SMM for maternal age younger than 14 years and older than 35 years.^27^ Although the number of previous pregnancies (ie, parity) could potentially confound the association of SMM with age, our subgroup analysis of only first-time mothers showed that the association of maternal age with SMM appears consistent for primiparous and multiparous women and adolescents. Associations between hospitals with the lowest pregnancy volume and SMM and maternal death were perhaps due to health care clinician volume of experience, such as the volume-outcomes association previously described in traumatic injury and ischemic cardiac disease.^28,29^

Body mass index and assisted reproductive technology (ART) use are known factors associated with SMM.^30,31^ Beyond age 40 years, conception is unlikely to happen without ART use. Hence, ART use may partly account for the greater risk of SMM among women 40 years or older in the present study, although these data were not reported in the CIHI DAD. Moreover, mothers 34 years or older in Canada have shown increased obstetric acute renal failure.^32^ Thus, baseline variables associated with aging (eg, lower cardiac output, hypertension, and atherosclerosis) could make older mothers vulnerable to significant physiological changes occurring during pregnancy, thereby increasing the risk of developing SMM. Moreover, placental insufficiency (ie, reduced uterine artery blood flow) is associated with preeclampsia.^33^ Increasing oxidative stress, an imbalance between free radical generation and antioxidant defense, with aging may be a key factor of placental insufficiency and subsequent development of SMM.^34^

### Clinical and Policy Relevance

While substantially lower than in the United States, Canada’s maternal mortality rate is greater than that in many high-income countries in Europe.^2^ Postpartum hemorrhage, cerebrovascular and cardiovascular disease related to pregnancy-induced hypertension, and sepsis are the most common underlying etiologies.^16^ Focusing on these causes and characteristics of SMM and mortality identified herein may be one mechanism to further reduce mortality for Canadian mothers. While not the most common causes of SMM, respiratory illness and cardiac illness are associated with a high risk of harm and death and should be an important focus for quality improvement initiatives, especially in older mothers (aged ≥35 years). Hemorrhage and sepsis are both more prevalent across all ages, despite a lower individual risk of harm or death, and are thus also important diagnoses as a focus of quality-of-care improvement. Because the absolute number of maternal deaths is very small, establishment of a program of surveillance and follow-up, such as the Confidential Enquiries Into Maternal Deaths in the United Kingdom,^35^ may be a feasible and appropriate approach. Compared with our results herein, the United Kingdom has a similar maternal mortality rate and accompanying increasing maternal age.^36^

The findings of an increasing risk of SMM and mortality with increasing maternal age may be one of many important public health and education considerations in the timing of pregnancy across the reproductive life span. Outreach to disadvantaged communities and individuals, early recognition and appropriate management of acute illness, facilitation of transfer to tertiary care for high-risk pregnant mothers, and interventions to detect and prevent progression of illness in at-risk mothers are important considerations for primary and specialist clinicians.^37,38^

### Limitations and Strengths

This study has some limitations. First, the CIHI DAD does not record certain variables that might influence maternal outcome (eg, body mass index, immigration status, race/ethnicity, frequency of visits to antenatal care, or validated measures of ART use).^30,31^ Second, although the DAD does not record deliveries that occur outside of hospitals, this represents only 2.1% of all deliveries in Canada.^39^ Third, the DAD does not identify deaths that have occurred outside of hospitals; however, this is rare for pregnant and postpartum women^40,41^ and is unlikely to have altered our findings. Fourth, 75% of therapeutic abortions normally occur in outpatient settings^42^ and are not captured in the DAD; however, subsequent complications of therapeutic abortions (eg, abortion-related hemorrhage and infection) requiring hospital admission are accounted for in our analyses.

Our study also has several strengths. First, this is the most contemporary nationwide population-based cohort study to date examining maternal outcomes in the context of increasing maternal age in Canada. Second, although most SMM occurred during the index delivery hospitalization and up to 6 weeks’ postpartum,^43^ creation of an episode of care enabled us to more sensitively capture SMM that occurred during the antepartum period. In the present study, 2.4% of all SMM events happened during the antepartum period, including ectopic pregnancies or abortions. Third, we used multilevel regression models to account for both patient-based and hospital-based factors associated with clinical outcomes, in addition to risk adjustment according to the Maternal Comorbidity Index.^17^ Sensitivity and subgroup analyses demonstrated our findings to be robust.^44,45,46,47^

## Conclusions

In Canada, maternal age and the incidence of SMM have increased over time. This study found that, province of residence, maternal comorbidity, income quintile, and extremes of maternal age, especially those 45 years and older compared with those aged 20 to 24 years, were associated with SMM and mortality. These findings have direct relevance for prospective parents, clinicians, and public health professionals.

## Notes

Supplement. **eTable 1.** List of variables in this study **eTable 2.** Frequency individual indicators of severe maternal morbidity, among 54,219 separate severe maternal morbidity events **eTable 3.** Most common causes of severe maternal morbidity, within the antepartum period, within the delivery or peripartum period, and within the postpartum readmission and the number needed to experience harm (death) for each condition (i.e. the number of women with this condition needed to result in one additional death) **eTable 4.** Most common causes of severe maternal morbidity by age category **eTable 5.** Estimated regression coefficients for the multi-level mixed logistic regression models for the outcome of Severe Maternal Morbidity [Outcome = Severe Maternal Morbidity, main predictor = quintile of hospitals according to the number of pregnancy admission at each hospital] **eTable 6.** Estimated regression coefficients for the multi-level mixed logistic regression models for the outcome of death [Outcome = death, main predictor = quintile of hospitals according to the number of pregnancy admission at each hospital] **eTable 7.** Sensitivity analysis: Estimated regression coefficients for the multi-level mixed logistic regression models with different main predictors for the outcome of SMM and death [Outcome = (a) Severe Maternal Morbidity, (b) Death, main predictors = quintile of hospitals according to (a) the number of pregnancy admission to ICU, (b) pregnancy-related ICU admission rate at each hospital] **eTable 8.** Sensitivity analysis: Estimated regression coefficients for the multi-level mixed logistic regression models for the outcome of Severe Maternal Morbidity, restricted to first pregnancy [Outcome = Severe Maternal Morbidity, main predictor = quintile of hospitals according to the number of pregnancy admission at each hospital] **eTable 9.** Estimated regression coefficients for the multi-level mixed logistic regression models for the outcome of (a) SMM, (b) Death within multiple imputation datasets [Outcome = (a) Severe Maternal Morbidity, (b) Death, main predictor = quintile of hospitals according to the number of pregnancy admission at each hospital] **eTable 10.** Sensitivity analysis: Estimated regression coefficients for the multi-level mixed logistic regression models for the outcome of Severe Maternal Morbidity, excluding those who had severe maternal morbidity during the episode of care and transfer on first hospital admission l during the episode [Outcome = severe maternal morbidity, main predictor = quintile of hospitals according to the number of pregnancy admission at each hospital]
