# Supplementary material for: Association of Maternal Age With Severe Maternal Morbidity and Mortality in Canada
Source: JAMA Netw Open. 2019 Aug 23;2(8):e199875. doi: 10.1001/jamanetworkopen.2019.9875 (PMC6714030; doi:10.1001/jamanetworkopen.2019.9875)
Supplement: Supplement. — eTable 1. List of variables in this study eTable 2. Frequency individual indicators of severe maternal morbidity, among 54,219 separate severe maternal morbidity events eTable 3. Most common causes of severe maternal morbidity, within the antepartum period, within the delivery or peripartum period, and within the postpartum readmission and the number needed to experience harm (death) for each condition (i.e. the number of women with this condition needed to result in one additional death) eTable 4. Most common causes of severe maternal morbidity by age category eTable 5. Estimated regression coefficients for the multi-level mixed logistic regression models for the outcome of Severe Maternal Morbidity [Outcome = Severe Maternal Morbidity, main predictor = quintile of hospitals according to the number of pregnancy admission at each hospital] eTable 6. Estimated regression coefficients for the multi-level mixed logistic regression models for the outcome of death [Outcome = death, main predictor = quintile of hospitals according to the number of pregnancy admission at each hospital] eTable 7. Sensitivity analysis: Estimated regression coefficients for the multi-level mixed logistic regression models with different main predictors for the outcome of SMM and death [Outcome = (a) Severe Maternal Morbidity, (b) Death, main predictors = quintile of hospitals according to (a) the number of pregnancy admission to ICU, (b) pregnancy-related ICU admission rate at each hospital] eTable 8. Sensitivity analysis: Estimated regression coefficients for the multi-level mixed logistic regression models for the outcome of Severe Maternal Morbidity, restricted to first pregnancy [Outcome = Severe Maternal Morbidity, main predictor = quintile of hospitals according to the number of pregnancy admission at each hospital] eTable 9. Estimated regression coefficients for the multi-level mixed logistic regression models for the outcome of (a) SMM, (b) Death within multiple imputation datasets [O [file jamanetwopen-2-e199875-s001.pdf]

Supplementary Online Content

Aoyama K, Pinto R, Ray JG, et al. Association of maternal age with severe maternal morbidity and mortality in Canada. *JAMA Netw Open*. 2019;2(8):e199875. doi:10.1001/jamanetworkopen.2019.9875

eTable 1. List of variables in this study
eTable 2. Frequency individual indicators of severe maternal morbidity, among 54,219 separate severe maternal morbidity events
eTable 3. Most common causes of severe maternal morbidity, within the antepartum period, within the delivery or peripartum period, and within the postpartum readmission and the number needed to experience harm (death) for each condition (i.e. the number of women with this condition needed to result in one additional death)
eTable 4. Most common causes of severe maternal morbidity by age category
eTable 5. Estimated regression coefficients for the multi-level mixed logistic regression models for the outcome of Severe Maternal Morbidity [Outcome = Severe Maternal Morbidity, main predictor = quintile of hospitals according to the number of pregnancy admission at each hospital]
eTable 6. Estimated regression coefficients for the multi-level mixed logistic regression models for the outcome of death [Outcome = death, main predictor = quintile of hospitals according to the number of pregnancy admission at each hospital]
eTable 7. Sensitivity analysis: Estimated regression coefficients for the multi-level mixed logistic regression models with different main predictors for the outcome of SMM and death [Outcome = (a) Severe Maternal Morbidity, (b) Death, main predictors = quintile of hospitals according to (a) the number of pregnancy admission to ICU, (b) pregnancy-related ICU admission rate at each hospital]
eTable 8. Sensitivity analysis: Estimated regression coefficients for the multi-level mixed logistic regression models for the outcome of Severe Maternal Morbidity, restricted to first pregnancy [Outcome = Severe Maternal Morbidity, main predictor = quintile of hospitals according to the number of pregnancy admission at each hospital]
eTable 9. Estimated regression coefficients for the multi-level mixed logistic regression models for the outcome of (a) SMM, (b) Death within multiple imputation datasets [Outcome = (a) Severe Maternal Morbidity, (b) Death, main predictor = quintile of hospitals according to the number of pregnancy admission at each hospital]
eTable 10. Sensitivity analysis: Estimated regression coefficients for the multi-level mixed logistic regression models for the outcome of Severe Maternal Morbidity, excluding those who had severe maternal morbidity during the episode of care and transfer on first hospital admission l during the episode [Outcome = severe maternal morbidity, main predictor = quintile of hospitals according to the number of pregnancy admission at each hospital]

This supplementary material has been provided by the authors to give readers additional information about their work.

© 2019 Aoyama K et al. *JAMA Network Open*.

eTable 1. List of variables in this study

Variable	Values
Patient-level variables	
Age in years [a]	Continuous
Age categories [a]	< 15 years, 15-19 years, 20-24 years, 25-29 years, 30-34 years, 35-39 years, 40-44 years, 45+ years
Maternal Comorbidity Index categories [a]	0, 1, $\geq$ 2
Parity categories [a]	0, 1, $\geq$ 2
Patient residence [a]	1 = Urban, 0 = Rural
Transfer status [b]	1 = Yes, 0 = No
Patient are income quintile [b]	1 (lowest), 2, 3, 4, 5 (highest)
Hospital-level variables	
Hospital location [b]	1 = Urban, 0 = Rural
Province of Hospital location [b]	Newfoundland and Labrador, Prince Edward Island, Nova Scotia, New Brunswick, Ontario, Manitoba, Saskatchewan, Alberta, British Columbia, Territories (Yukon, Northwest Territories and Nunavut)
Number of pregnancy related hospital admission	Continuous
Hospital volume categories [a]	0 (less than 55 over the study period), 1 (lowest), 2, 3, 4, 5 (highest)
Outcome variables	
ICU admission [b]	1 = Yes, 0 = No
Death [b]	1 = Yes, 0 = No
Severe Maternal Morbidity [b]	1 = Yes, 0 = No

a: measured at first hospital admission over an entire pregnancy episode
b: measured at any hospital admission over an entire pregnancy episode

© 2019 Aoyama K et al. *JAMA Network Open*.

eTable 2. Frequency individual indicators of severe maternal morbidity, among 54,219 separate severe maternal morbidity events[a]

**Indicator of several maternal morbidity**	**No. (%)**
Septicemia during labour, Puerperal sepsis	12,011 (0.38)
Cardiac arrest, cardiac failure, myocardial infarction or pulmonary edema	4,901 (0.15)
Placental abruption with coagulation defect, Antepartum hemorrhage with coagulation defect, Intrapartum hemorrhage with coagulation defect, Rupture of uterus before onset of labour, or during labour	4,133 (0.13)
Eclampsia	2,604 (0.08)
Obstetric embolism	1,783 (0.06)
Acute abdomen	1,708 (0.05)
Acute Renal Failure	1,296 (0.04)
Obstetric shock	1,285 (0.04)
Cardiomyopathy in the puerperium	1,124 (0.04)
Acute psychosis	740 (0.02)
Pulmonary, cardiac and central nervous system complications of anaesthesia during pregnancy, labour and delivery and the puerperium	416 (0.01)
Cerebrovascular diseases: subarachnoid and intracranial hemorrhage, cerebral infarction, stroke	394 (0.01)
Disseminated intravascular coagulation	327 (0.01)
Adult respiratory distress syndrome	317 (0.01)
Sickle cell anemia with crisis	189 (0.01)
Hepatic failure	144 (0.00)
Cerebral venous thrombosis in pregnancy & the puerperium	131 (0.00)
Status asthmaticus	131 (0.00)
Status epilepticus	120 (0.00)
Pre-existing hypertensive heart disease, Pre-existing hypertensive heart and renal disease	92 (0.00)
Cerebral edema or coma	75 (0.00)
Death -- obstetric, cause unspecified; death, obstetric, after 42 days but 1 year after delivery; death from sequelae of direct obstetric causes; or sudden death, death from unspecified cause	41 (0.00)
Death of any causes	178 (0.00)
HIV	1,634 (0.05)
**Canadian Classification of Health Interventions code-related severe maternal morbidity**	

© 2019 Aoyama K et al. *JAMA Network Open*.

**variables**	
Blood transfusion (red blood cell, plasma, platelet, albumin or others)	48,473 (1.5)
Postpartum hemorrhage + blood transfusion	17,456 (0.55)
Embolization or ligation of pelvic vessels or suturing of uterus + postpartum hemorrhage	3,288 (0.10)
Subtotal hysterectomy, open approach	3,006 (0.10)
Assisted ventilation through endotracheal tube, assisted ventilation through tracheostomy	2,425 (0.08)
Repair of bladder, urethra or intestine	2,122 (0.07)
Postpartum hemorrhage + hysterectomy	1,731 (0.05)
Placenta previa with hemorrhage + blood transfusion	1,528 (0.05)
Evacuation of incisional hematoma	1,085 (0.03)
Total hysterectomy, open approach	910 (0.03)
Intrapartum hemorrhage + blood transfusion	802 (0.03)
Caesarean hysterectomy	784 (0.02)
Dialysis	227 (0.01)

[a]: Categories are not mutually exclusive

© 2019 Aoyama K et al. *JAMA Network Open*.

eTable 3. Most common causes of severe maternal morbidity, within the antepartum period, within the delivery or peripartum period, and within the postpartum readmission and the number needed to experience harm (death) for each condition (i.e. the number of women with this condition needed to result in one additional death)[a]

Timing		No. (%)	NNEH
**Antepartum**	Acute abdomen [b]	108 (0.90)	215.2
	Obstetric embolism [c]	31 (0.26)	60.7
	Cardiac arrest, cardiac failure, myocardial infarction or pulmonary edema	30 (0.25)	27.2
	Need for mechanical ventilation [d]	24 (0.20)	15.2
	Sepsis	13 (0.11)	13.0
**Delivery or peripartum**	Postpartum hemorrhage and receive blood transfusion	14,226 (0.48)	390.8
	Placental abruption with coagulation defect; antepartum hemorrhage with coagulation defect; Intrapartum hemorrhage with coagulation defect; Rupture of uterus before onset of labour, or during labour	3845 (0.13)	311.8
	Cardiac arrest, cardiac failure, myocardial infarction or pulmonary edema	3589 (0.12)	49.8
	Sepsis	2928 (0.10)	625.1
	Embolization or ligation of pelvic vessels or suturing of uterus and postpartum hemorrhage	2808 (0.09)	501.3
	Subtotal hysterectomy, open approach	2700 (0.09)	250.5
**Postpartum readmission**	Sepsis	9065 (14.8)	964.4
	Postpartum hemorrhage and blood transfusion	3176 (5.2)	298.7
	Cardiac arrest, cardiac failure, myocardial infarction or pulmonary edema	1166 (1.9)	41.2
	Need for mechanical ventilation [d]	694 (1.1)	11.4
	Obstetric embolism [c]	625 (1.0)	110.4

[a]: Categories are not mutually exclusive
[b]: Acute abdomen: acute appendicitis, peritonitis, female acute pelvic peritonitis
[c]: Obstetric embolism: obstetric air embolism, blood-clot embolism, septic embolism and amniotic fluid embolism
[d]: Need for mechanical ventilation: assisted ventilation through endotracheal tube, assisted ventilation through tracheostomy
NNEH: Number needed to experience harm: the number of women with this condition needed to result in one additional death

© 2019 Aoyama K et al. *JAMA Network Open*.

eTable 4. Most common causes of severe maternal morbidity by age category[a]

Age category		Counts (rate per 1000 deliveries)
**Adolescent (10- to 19-year-old)**	Postpartum hemorrhage and receive blood transfusion	1,043 (7.5)
	Sepsis	889 (6.4)
	Eclampsia	213 (1.5)
**20- to 34-year-old**	Postpartum hemorrhage and receive blood transfusion	11,881 (4.9)
	Sepsis	8,570 (3.6)
	Cardiac arrest, cardiac failure, myocardial infarction or pulmonary edema	2,958 (1.2)
**Older mothers (35-year-old or older)**	Postpartum hemorrhage and receive blood transfusion	3,440 (5.6)
	Sepsis	2,098 (3.4)
	Cardiac arrest, cardiac failure, myocardial infarction or pulmonary edema	1,415 (2.3)

[a]: Categories are not mutually exclusive

© 2019 Aoyama K et al. *JAMA Network Open*.

eTable 5. Estimated regression coefficients for the multi-level mixed logistic regression models for the outcome of Severe Maternal Morbidity [Outcome=Severe Maternal Morbidity, main predictor= quintile of hospitals according to the number of pregnancy admission at each hospital]

Variable	Final model for Severe Maternal Morbidity with Hospital group according to Hospital pregnancy volume	
	Regression coefficient (95% CI)	P-value
Intercept	-4.86 (-5.04, -4.67)	<0.0001
Patient variables		
Maternal Comorbidity Index 0	Reference	
1	0.48 (0.45, 0.51)	<0.0001
> 1	1.3 (1.27, 1.33)	<0.0001
Age < 15	0.17 (-0.25, 0.58)	0.4287
15-19	0.13 (0.08, 0.18)	<0.0001
20-24	Reference	
25-29	0.01 (-0.02, 0.04)	0.4266
30-34	0.11 (0.08, 0.14)	<0.0001
35-39	0.27 (0.24, 0.31)	<0.0001
40-44	0.47 (0.42, 0.52)	<0.0001
45+	0.99 (0.85, 1.12)	<0.0001
Parity 0	0.26 (0.23, 0.29)	<0.0001
1	-0.09 (-0.12, -0.06)	<0.0001
> 1	Reference	
Residence (urban/rural)	0.02 (-0.01, 0.05)	0.1437
Transfer	0.7 (0.64, 0.76)	<0.0001
Income quintile		
1 (lowest)	0.18 (0.15, 0.21)	<0.0001
2	0.11 (0.08, 0.14)	<0.0001
3	0.05 (0.02, 0.08)	0.0033
4	0.02 (-0.02, 0.05)	0.2828
5 (highest)	Reference	
Hospital variables		
Groups according to Hospital volume of pregnancy		
1 (lowest volume)	0.19 (0.01, 0.38)	0.0427
2	0.06 (-0.08, 0.21)	0.3947
3	0.13 (0.01, 0.26)	0.041
4	0.04 (-0.09, 0.16)	0.5381
5 (highest volume)	Reference	
Province		
Newfoundland and Labrador	0.11 (-0.12, 0.34)	0.3482
Prince Edward Island	0.07 (-0.43, 0.58)	0.7829
Nova Scotia	-0.01 (-0.25, 0.24)	0.9585
New Brunswick	-0.08 (-0.31, 0.15)	0.504
Ontario	Reference	
Manitoba	0.05 (-0.15, 0.25)	0.6112
Saskatchewan	0.19 (0.01, 0.38)	0.0396
Alberta	0.15 (0.02, 0.28)	0.0192
British Columbia	-0.25 (-0.38, -0.12)	0.0001
Territories	0.54 (0, 1.08)	0.0512
Hospital (Urban/rural)	0.1 (-0.05, 0.25)	0.1889

SMM: Severe Maternal Morbidity

© 2019 Aoyama K et al. *JAMA Network Open*.

eTable 6. Estimated regression coefficients for the multi-level mixed logistic regression models for the outcome of death [Outcome=death, main predictor= quintile of hospitals according to the number of pregnancy admission at each hospital]

Variable	Final model for death with Hospital group according to Hospital pregnancy volume	
	Regression coefficient (95% CI)	P-value
Intercept	-13.13 (-14.93, -11.33)	<0.0001
Patient variables		
Maternal Comorbidity Index		
0	Reference	
1	0.53 (0.06, 1)	0.0274
> 1	2.32 (2.32, 2.32)	<0.0001
Age		
< 15	-12.24 (-3543.49, 3519.01)	0.9946
15-19	-0.55 (-1.63, 0.52)	0.3117
20-24	Reference	
25-29	0.23 (-0.29, 0.76)	0.3822
30-34	0.23 (-0.3, 0.76)	0.3944
35-39	0.66 (0.1, 1.22)	0.0211
40-44	1.22 (0.52, 1.92)	0.0006
45+	1.48 (0.01, 2.95)	0.0491
Parity		
0	0.47 (0, 0.94)	0.0495
1	0.21 (-0.29, 0.72)	0.4077
> 1	Reference	
Residence (urban/rural)	0.01 (-0.43, 0.45)	0.975
Transfer	2.4 (1.96, 2.84)	<0.0001
Income quintile		
1 (lowest)	1.42 (0.71, 2.14)	0.0001
2	1.22 (0.49, 1.95)	0.0011
3	1.37 (0.64, 2.09)	0.0002
4	1.1 (0.35, 1.85)	0.0041
5 (highest)	Reference	
Hospital variables		
Groups according to Hospital volume of pregnancy		
1 (lowest volume)	1.66 (0.55, 2.77)	0.0033
2	0.45 (-0.65, 1.55)	0.4216
3	Reference	
4	0 (-0.76, 0.75)	0.9945
5 (highest volume)	0.17 (-0.53, 0.86)	0.6436
Province		
Newfoundland and Labrador	0.3 (-0.69, 1.29)	0.5502
Nova Scotia	-0.28 (-1.28, 0.73)	0.5891
New Brunswick	-0.99 (-2.43, 0.46)	0.18
Ontario	Reference	
Manitoba	-0.43 (-1.24, 0.38)	0.3002
Saskatchewan	0.08 (-0.61, 0.78)	0.8122
Alberta	-0.29 (-0.78, 0.19)	0.2377
British Columbia	-0.73 (-1.29, -0.18)	0.0098
Hospital (Urban/rural)	1.04 (-0.43, 2.51)	0.1653

© 2019 Aoyama K et al. *JAMA Network Open*.

eTable 7. Sensitivity analysis: Estimated regression coefficients for the multi-level mixed logistic regression models with different main predictors for the outcome of SMM and death [Outcome=(a) Severe Maternal Morbidity, (b) Death, main predictors= quintile of hospitals according to (a) the number of pregnancy admission to ICU, (b) pregnancy-related ICU admission rate at each hospital]

	Final models for Severe Maternal Morbidity				Final models for death			
	(a) with Hospital group according to pregnancy-related ICU volume		(b) with Hospital group according to pregnancy-related ICU admission rate		(a) with Hospital group according to pregnancy-related ICU volume		(b) with Hospital group according to pregnancy-related ICU admission rate	
	Regression coefficient (95% CI)	P-value	Regression coefficient (95% CI)	P-value	Regression coefficient (95% CI)	P-value	Regression coefficient (95% CI)	P-value
Intercept	-4.91 (-5.1, -4.71)	<0.0001	-4.89 (-5.08, -4.71)	<0.0001	-90.63 (-93.1, -88.16)	<0.0001	-29.22 (-31.47,-26.96)	<0.0001
Patient variables								
Maternal Comorbidity Index								
0	Reference		Reference		Reference		Reference	
1	0.48 (0.45, 0.51)	<0.0001	0.48 (0.45, 0.51)	<0.0001	0.51 (0.04, 0.98)	0.0343	0.51 (0.04, 0.98)	0.0326
> 1	1.3 (1.27, 1.33)	<0.0001	1.3 (1.27, 1.33)	<0.0001	2.28 (1.93, 2.63)	<0.0001	2.26 (1.92, 2.61)	<0.0001
Age								
< 15	0.17 (-0.25, 0.58)	0.4272	0.17 (-0.25, 0.59)	0.4252	Reference		Reference	
15-19	0.13 (0.08, 0.18)	<0.0001	0.13 (0.09, 0.18)	<0.0001	77.07 (75.35, 78.79)	<0.0001	15.5 (13.78, 17.22)	<0.0001
20-24	Reference				77.61 (76.14, 79.07)	<0.0001	16.05 (14.59, 17.52)	<0.0001
25-29	0.01 (-0.02, 0.04)	0.4458	0.01 (-0.02, 0.04)	0.4535	77.83 (76.39, 79.26)	<0.0001	16.29 (14.86, 17.72)	<0.0001
30-34	0.11 (0.08, 0.14)	<0.0001	0.11 (0.08, 0.14)	<0.0001	77.82 (76.39, 79.25)	<0.0001	16.29 (14.86, 17.72)	<0.0001
35-39	0.27 (0.24, 0.31)	<0.0001	0.27 (0.23, 0.31)	<0.0001	78.25 (76.81, 79.68)	<0.0001	16.72 (15.29, 18.16)	<0.0001
40-44	0.47 (0.42, 0.52)	<0.0001	0.47 (0.42, 0.52)	<0.0001	78.81 (77.32, 80.3)	<0.0001	17.27 (15.78, 18.76)	<0.0001
45+	0.99 (0.85, 1.12)	<0.0001	0.98 (0.85, 1.12)	<0.0001	79.11 (0, 0)	<0.0001	17.56 (0, 0)	<0.0001
Parity								
0	0.26 (0.23, 0.29)	<0.0001	0.26 (0.23, 0.29)	<0.0001	0.46 (-0.01, 0.93)	0.0543	0.47 (0, 0.94)	0.0513
1	-0.09 (-0.12, -0.06)	<0.0001	-0.09 (-0.12, -0.06)	<0.0001	0.2 (-0.3, 0.71)	0.4316	0.21 (-0.3, 0.72)	0.4158
> 1	Reference		Reference		Reference		Reference	
Residence (urban/rural)	0.02 (-0.01, 0.05)	0.2022	0.02 (-0.01, 0.04)	0.2394	-0.07 (-0.5, 0.36)	0.7435	0 (-0.42, 0.42)	0.9995
Transfer	0.71 (0.65, 0.77)	<0.0001	0.71 (0.65, 0.77)	<0.0001	2.53 (2.1, 2.96)	<0.0001	2.48 (2.06, 2.91)	<0.0001
Income quintile								
1 (lowest)	0.17 (0.13, 0.2)	<0.0001	0.17 (0.13, 0.2)	<0.0001	1.42 (0.71, 2.14)	0.0001	1.43 (0.71, 2.14)	0.0001
2	0.1 (0.07, 0.14)	<0.0001	0.1 (0.07, 0.14)	<0.0001	1.2 (0.47, 1.94)	0.0014	1.21 (0.48, 1.95)	0.0012
3	0.05 (0.01, 0.08)	0.006	0.05 (0.01, 0.08)	0.0063	1.35 (0.62, 2.08)	0.0003	1.37 (0.64, 2.1)	0.0002
4	0.02 (-0.02, 0.05)	0.3157	0.02 (-0.02, 0.05)	0.3271	1.09 (0.34, 1.84)	0.0044	1.11 (0.36, 1.86)	0.0037
5 (highest)	Reference		Reference		Reference		Reference	

© 2019 Aoyama K et al. *JAMA Network Open*.

Hospital variables								
Groups according to Hospital volume of pregnancy								
1 (lowest volume)	Reference		Reference		0.54 (-1.18, 2.26)	0.5383	0.44 (-0.69, 1.57)	0.4439
2	0.14 (-0.03, 0.32)	0.1148	0.1 (-0.06, 0.26)	0.2172	Reference		Reference	
3	0.25 (0.09, 0.41)	0.0021	0.06 (-0.09, 0.21)	0.4257	0.23 (-1.35, 1.81)	0.7715	0.11 (-0.63, 0.86)	0.7614
4	0.13 (-0.03, 0.29)	0.1121	0.11 (-0.04, 0.26)	0.1421	0.76 (-0.73, 2.24)	0.3184	0.58 (-0.15, 1.32)	0.1204
5 (highest volume)	0.17 (0.01, 0.32)	0.0364	0.38 (0.23, 0.54)	<0.0001	0.75 (-0.72, 2.21)	0.317	0.86 (0.12, 1.6)	0.0224
Province								
Newfoundland and Labrador	0.12 (-0.12, 0.36)	0.3226	0.02 (-0.21, 0.25)	0.8805	0.34 (-0.64, 1.33)	0.4951	0.11 (-0.84, 1.06)	0.8227
Prince Edward Island	0.03 (-0.48, 0.55)	0.8989	0.17 (-0.33, 0.66)	0.5051	Not included*		Not included*	
Nova Scotia	-0.02 (-0.27, 0.23)	0.8788	0.06 (-0.18, 0.29)	0.6494	-0.22 (-1.24, 0.8)	0.6695	0.08 (-0.91, 1.07)	0.8781
New Brunswick	-0.02 (-0.26, 0.21)	0.85	-0.05 (-0.28, 0.17)	0.6538	-1.02 (-2.48, 0.43)	0.1681	-0.89 (-2.32, 0.54)	0.2201
Ontario	Reference		Reference		Reference		Reference	
Manitoba	0.1 (-0.1, 0.31)	0.3295	0.16 (-0.04, 0.36)	0.1234	-0.33 (-1.16, 0.5)	0.4376	-0.12 (-0.92, 0.67)	0.7592
Saskatchewan	0.23 (0.04, 0.42)	0.0171	0.25 (0.07, 0.43)	0.0056	0.18 (-0.52, 0.88)	0.6173	0.21 (-0.45, 0.87)	0.5366
Alberta	0.23 (0.09, 0.37)	0.001	0.27 (0.13, 0.4)	0.0001	-0.23 (-0.73, 0.28)	0.3794	-0.01 (-0.51, 0.49)	0.9682
British Columbia	-0.22 (-0.36, -0.09)	0.0012	-0.19 (-0.32, -0.07)	0.0031	-0.73 (-1.3, -0.15)	0.0129	-0.51 (-1.06, 0.03)	0.066
Territories	0.48 (-0.07, 1.03)	0.0879	0.59 (0.07, 1.12)	0.0272	Not included [a]		Not included [a]	
Hospital (Urban/rural)	0.04 (-0.11, 0.19)	0.6066	0.02 (-0.12, 0.16)	0.7667	0.45 (-1.03, 1.93)	0.5526	0.67 (-0.77, 2.11)	0.3596

[a]: Because of no death or only one death during the study period, this province was not included in the analyses.

© 2019 Aoyama K et al. *JAMA Network Open*.

eTable 8. Sensitivity analysis: Estimated regression coefficients for the multi-level mixed logistic regression models for the outcome of Severe Maternal Morbidity, restricted to first pregnancy [Outcome=Severe Maternal Morbidity, main predictor= quintile of hospitals according to the number of pregnancy admission at each hospital]

Variable	Final model for Severe Maternal Morbidity, restricted to first pregnancy	
	Regression coefficient (95% CI)	P-value
Intercept	-4.52 (-4.71, -4.33)	<0.0001
Patient variables		
Maternal Comorbidity Index 0	Reference	
1	0.44 (0.4, 0.48)	<0.0001
> 1	1.29 (1.26, 1.33)	<0.0001
Age < 15	0.17 (-0.25, 0.6)	0.4217
15-19	0.13 (0.08, 0.19)	<0.0001
20-24	Reference	
25-29	0.02 (-0.02, 0.05)	0.3869
30-34	0.13 (0.09, 0.17)	<0.0001
35-39	0.28 (0.23, 0.32)	<0.0001
40-44	0.5 (0.44, 0.57)	<0.0001
45+	1.12 (0.96, 1.28)	<0.0001
Parity 0	0.26 (0.23, 0.29)	<0.0001
1	-0.09 (-0.12, -0.06)	<0.0001
> 1	Reference	
Residence (urban/rural)	0.03 (0, 0.07)	0.0825
Transfer	0.6 (0.53, 0.68)	<0.0001
Income quintile		
1 (lowest)	0.1 (0.06, 0.14)	<0.0001
2	0.08 (0.04, 0.12)	0.0003
3	0.02 (-0.03, 0.06)	0.4374
4	0 (-0.04, 0.05)	0.8664
5 (highest)	Reference	
Hospital variables		
Groups according to Hospital volume of pregnancy		
1 (lowest volume)	0.33 (0.12, 0.53)	0.002
2	0.1 (-0.05, 0.26)	0.1886
3	0.16 (0.04, 0.29)	0.0112
4	0.04 (-0.08, 0.17)	0.4662
5 (highest volume)	Reference	
Province		
Newfoundland and Labrador	0.04 (-0.18, 0.27)	0.7195
Prince Edward Island	0.04 (-0.44, 0.52)	0.8762
Nova Scotia	-0.05 (-0.29, 0.18)	0.6622
New Brunswick	-0.13 (-0.35, 0.09)	0.2617
Ontario	Reference	
Manitoba	0.05 (-0.16, 0.25)	0.6418
Saskatchewan	0.14 (-0.05, 0.34)	0.1491
Alberta	0.11 (-0.02, 0.25)	0.092
British Columbia	-0.31 (-0.44, -0.18)	0.0001
Territories	0.31 (-0.28, 0.9)	0.3066
Hospital (Urban/rural)	0.08 (-0.09, 0.24)	0.3586

© 2019 Aoyama K et al. *JAMA Network Open*.

eTable 9. Estimated regression coefficients for the multi-level mixed logistic regression models for the outcome of (a) SMM, (b) Death within multiple imputation datasets [Outcome=(a) Severe Maternal Morbidity, (b) Death, main predictor= quintile of hospitals according to the number of pregnancy admission at each hospital]

Variable	Final model for Severe Maternal Morbidity		Final model for death	
	Regression coefficient (95% CI)	P-value	Regression coefficient (95% CI)	P-value
Intercept	-4.8 (-4.97, -4.63)	<0.0001	-12.62 (-12.84, -12.44)	<0.0001
Patient variables				
Maternal Comorbidity Index 0	Reference		Reference	
1	0.49 (0.46, 0.51)	<0.0001	0.50 (0.48, 0.52)	0.0274
> 1	1.31 (1.28, 1.33)	<0.0001	2.32 (2.32, 2.32)	<0.0001
Age				
< 15	0.29 (-0.07, 0.66)	0.1139	-14.19 (-19.31, -10.39)	0.9827
15-19	0.13 (0.08, 0.17)	<0.0001	-0.2 (-0.21, -0.19)	0.6677
20-24	Reference		Reference	
25-29	0.01 (-0.02, 0.03)	0.7296	0.32 (0.31, 0.33)	0.2129
30-34	0.1 (0.07, 0.13)	<0.0001	0.18 (0.16, 0.2)	0.4944
35-39	0.27 (0.24, 0.31)	<0.0001	0.67 (0.64, 0.69)	0.0146
40-44	0.47 (0.42, 0.52)	<0.0001	1.2 (1.18, 1.22)	0.0004
45+	0.96 (0.83, 1.08)	<0.0001	1.5 (1.47, 1.53)	0.0444
Parity 0	0.28 (0.25, 0.3)	<0.0001	0.46 (0.44, 0.48)	0.0495
1	-0.1 (-0.13, -0.07)	<0.0001	0.20 (0.18, 0.22)	0.4077
> 1	Reference		Reference	
Residence (urban/rural)	0.01 (-0.02, 0.03)	0.5326	-0.06 (-0.07, -0.04)	0.7922
Transfer	0.69 (0.64, 0.75)	<0.0001	2.46 (2.46, 2.47)	<0.0001
Income quintile				
1 (lowest)	0.17 (0.14, 0.2)	<0.0001	1.4 (1.19, 1.66)	0.0001
2	0.1 (0.07, 0.14)	<0.0001	1.15 (0.92, 1.38)	0.0019
3	0.05 (0.01, 0.08)	0.0057	1.27 (1.04, 1.45)	0.0005
4	0.01 (-0.02, 0.05)	0.4606	1.01 (0.84, 1.29)	0.0066
5 (highest)	Reference		Reference	
Hospital variables				
Groups according to Hospital volume of pregnancy				
1 (lowest volume)	0.18 (0, 0.37)	0.0533	1.66 (1.65, 1.67)	0.0015
2	0.08 (-0.07, 0.22)	0.2865	0.35 (0.34, 0.36)	0.5248
3	0.13 (0.01, 0.26)	0.0401	Reference	
4	0.04 (-0.08, 0.16)	0.5287	-0.02 (-0.03, -0.02)	0.9468
5 (highest volume)	Reference		0.1 (0.09, 0.1)	0.7664
Province				
Newfoundland and Labrador	0.09 (-0.15, 0.32)	0.4634	0.3 (0.3, 0.31)	0.5304
Prince Edward Island	0.05 (-0.47, 0.56)	0.8625	Not included*	
Nova Scotia	-0.01 (-0.26, 0.23)	0.9062	-0.51 (-0.52, -0.5)	0.3005
New Brunswick	-0.11 (-0.34, 0.13)	0.3715	-1.01 (-1.01, -1)	0.1667
Ontario			Reference	
Manitoba	0.04 (-0.16, 0.24)	0.6834	-0.33 (-0.33, -0.33)	0.3887
Saskatchewan	0.17 (-0.01, 0.36)	0.0701	-0.01 (-0.02, -0.01)	0.9666
Alberta	0.12 (0, 0.25)	0.0595	-0.32 (-0.32, -0.31)	0.1541
British Columbia	-0.26 (-0.39, -0.13)	0.0001	-0.6 (-0.61, -0.59)	0.0154
Territories	0.34 (-0.06, 0.74)	0.0994	Not included*	

© 2019 Aoyama K et al. *JAMA Network Open*.

Hospital (Urban/rural)	0.06 (-0.07, 0.19)	0.3845	1.23 (1.22, 1.24)	0.0983

© 2019 Aoyama K et al. *JAMA Network Open*.

eTable 10. Sensitivity analysis: Estimated regression coefficients for the multi-level mixed logistic regression models for the outcome of Severe Maternal Morbidity, excluding those who had severe maternal morbidity during the episode of care and transfer on first hospital admission l during the episode [Outcome=severe maternal morbidity, main predictor= quintile of hospitals according to the number of pregnancy admission at each hospital]

Variable	Final model for Severe Maternal Morbidity, excluding those who had severe maternal morbidity before the transfer at first admission hospital	
	Regression coefficient (95% CI)	P-value
Intercept	-4.86 (-5.04, -4.67)	<0.0001
Maternal Comorbidity Index 0	Reference	
1	0.48 (0.46, 0.51)	<0.0001
> 1	1.31 (1.28, 1.34)	<0.0001
Age < 15	0.14 (-0.29, 0.56)	0.5324
15-19	0.13 (0.08, 0.18)	<0.0001
20-24	Reference	
25-29	0.01 (-0.02, 0.04)	0.4721
30-34	0.11 (0.07, 0.14)	<0.0001
35-39	0.27 (0.23, 0.3)	<0.0001
40-44	0.47 (0.42, 0.53)	<0.0001
45+	0.98 (0.85, 1.12)	<0.0001
Parity 0	0.26 (0.23, 0.29)	<0.0001
1	-0.09 (-0.12, -0.06)	<0.0001
> 1	Reference	
Residence (urban/rural)	0.02 (-0.01, 0.05)	0.1292
Transfer	0.5 (0.43, 0.56)	<0.0001
Income quintile		
1 (lowest)	0.17 (0.13, 0.2)	<0.0001
2	0.1 (0.07, 0.14)	<0.0001
3	0.05 (0.01, 0.08)	0.0056
4	0.02 (-0.02, 0.05)	0.2914
5 (highest)	Reference	
Groups according to Hospital volume of pregnancy		
1 (lowest volume)	0.2 (0.01, 0.39)	0.0346
2	0.08 (-0.06, 0.22)	0.2828
3	0.14 (0.02, 0.27)	0.0252
4	0.04 (-0.08, 0.16)	0.4963
5 (highest volume)	Reference	
Province		
Newfoundland and Labrador	0.11 (-0.12, 0.33)	0.3498
Prince Edward Island	0.08 (-0.41, 0.57)	0.7561
Nova Scotia	-0.04 (-0.28, 0.2)	0.7331
New Brunswick	-0.08 (-0.3, 0.14)	0.4733
Ontario	Reference	
Manitoba	0.06 (-0.13, 0.26)	0.5146
Saskatchewan	0.19 (0.01, 0.37)	0.0413
Alberta	0.16 (0.03, 0.28)	0.0123
British Columbia	-0.25 (-0.38, -0.13)	0.0001
Territories	0.54 (0.02, 1.07)	0.0434
Hospital (Urban/rural)	0.1 (-0.05, 0.24)	0.196

© 2019 Aoyama K et al. *JAMA Network Open*.
